# The Adipose Tissue Macrophages Central to Adaptive Thermoregulation

**DOI:** 10.3389/fimmu.2022.884126

**Published:** 2022-04-12

**Authors:** Md. Shamim Rahman, Heejin Jun

**Affiliations:** Department of Nutritional Sciences, College of Human Sciences, Texas Tech University, Lubbock, TX, United States

**Keywords:** adipose tissue macrophage, beige adipocyte, brown adipocyte, obesity, thermogenesis

## Abstract

White fat stores excess energy, and thus its excessive expansion causes obesity. However, brown and beige fat, known as adaptive thermogenic fat, dissipates energy in the form of heat and offers a therapeutic potential to counteract obesity and metabolic disorders. The fat type-specific biological function is directed by its unique tissue microenvironment composed of immune cells, endothelial cells, pericytes and neuronal cells. Macrophages are major immune cells resident in adipose tissues and gained particular attention due to their accumulation in obesity as the primary source of inflammation. However, recent studies identified macrophages’ unique role and regulation in thermogenic adipose tissues to regulate energy expenditure and systemic energy homeostasis. This review presents the current understanding of macrophages in thermogenic fat niches with an emphasis on discrete macrophage subpopulations central to adaptive thermoregulation.

## Introduction

Obesity is directly linked to the onset of many diseases, including type 2 diabetes, hypertension, cardiovascular diseases and some types of cancer (1, 2). Therefore, weight loss is suggested as a fundamental approach to ameliorate those disorders (3, 4). Obesity develops when energy intake chronically exceeds energy expenditure, and adipose tissue is the center of the regulation of systemic energy homeostasis. An expansion of white adipose tissue (WAT) in which energy-storing white adipocytes grow in number (hyperplasia) and size (hypertrophy) is the major characteristic of obesity (5). Conversely, brown adipocytes found in brown adipose tissue (BAT) and beige adipocytes inducible in particular WAT depots dissipate excess energy as heat, termed adaptive thermogenesis (5, 6). Since the biological functions of thermogenic adipocytes include regulating lipid and glucose metabolism and insulin sensitivity beyond strengthened energy expenditure, enhancing thermogenic adipocyte activity and amount holds promise to combat obesity and related disorders (7–10).

The distinct biological functions of white and thermogenic adipocytes are directed by their unique tissue microenvironments composed of multiple cell types, including immune cells, adipocyte progenitors, endothelial cells, pericytes and neuronal cells (11). In particular, immune cells have received considerable attention owing to their role in WAT inflammation, key pathophysiology of obesity. Macrophages are the most plentiful WAT-infiltrating immune cells in obesity and are crucial mediators of inflammation in adipose tissues (12). Adipose tissue macrophages (ATMs) are a heterogeneous population but can be conventionally classified as M1 (classically activated or pro-inflammatory) or M2 (anti-inflammatory or alternatively activated) cells. In obese WAT, M1 macrophages are recruited in response to pro-inflammatory mediators (lipopolysaccharides, interferon-gamma among others) and release pro-inflammatory cytokines [tumor necrosis factor-alpha (TNFα), inducible nitric oxide synthase) (13). Oppositely, M2 macrophages are abundantly found in lean WAT, express specific biomarkers (e.g., arginase) and secrete anti-inflammatory cytokines [e.g., transforming growth factor-beta (TGFβ), interleukin (IL)-10, IL-1 decoy receptor] (13).

In contrast to WAT, there was a limited research interest in the role of ATMs in regulating thermogenic adipose tissue characteristics and functions. The accumulation of pro-inflammatory M1 macrophages is observed in thermogenic adipose tissues in the state of obese and impairs thermogenic machinery of brown and beige adipocytes (14). Intriguingly, recent studies identified and highlighted the non-canonical roles of ATMs found in thermogenic adipose niches. Certain ATM subsets are specialized to mediate thermogenic programs of brown or beige adipocytes through driving local noradrenergic or cholinergic tone (15–20). This review covers the roles of ATMs in thermogenic adipose tissues, emphasizing distinct macrophage subpopulations central to adaptive thermoregulation.

## A Distinct Role of Thermogenic Fat in Energy Homeostasis

In humans, thermogenic fat was believed to exist restrictively to infancy to generate heat and fight against hypothermia under cold environments. However, in 2009 a series of milestone publications identified the presence of functional thermogenic fat in the upper supraclavicular region of adult humans (21–23). Thermogenic activity, determined by 18F-fluorodeoxyglucose uptake, was induced in the supraclavicular area following short cold exposure, and its biopsy specimens showed the molecular and morphological features of thermogenic fat. So far, adult humans have been known to have thermogenic fat in several anatomic areas, including cervical, axillary, paraspinal, mediastinal, and abdominal, as well as supraclavicular (24). A recent study implied the significance of thermogenic fat in humans by demonstrating a lower prevalence of cardiometabolic disorders, including type 2 diabetes, coronary artery disease, hypertension and congestive heart failure, in individuals with thermogenic fat (25).

To date, three types of adipocytes have been identified in mammals: white, brown and beige adipocytes. While white adipocytes found in WAT possess few mitochondria and a large unilocular lipid droplet to store energy efficiently, brown and beige adipocytes contain multilocular lipid droplets and numerous mitochondria to generate heat. However, despite their similarities, brown and beige adipocytes are distinct cell types due to the main differences in their anatomical location and developmental origin (26). Therefore, brown adipocytes are specified as “classic” in distinction from beige adipocytes. Classic brown adipocytes cluster in dedicated depots, such as interscapular BAT of infants and mice. Thermogenic function of brown adipocytes is active at birth and relatively stable because they develop prenatally from precursors in the dermomyotome that expresses myogenic factor 5 (*Myf5*), paired-box protein 7 (*Pax7*) and engrailed 1 (27–29). However, beige adipocytes are inducible in WAT, particularly subcutaneous WAT, in response to cold exposure, catecholamines, thiazolidinediones and exercise. They derive from *Myf5*-absent precursors during postnatal development in mice, and upon cold exposure, beige adipocytes can be recruited by differentiation of precursors expressing alpha-smooth muscle actin (*αSma*), *Cd81, Pax3* or platelet-derived growth factor receptor, alpha polypeptide (*Pdgfrα*) (30–33).

The long-standing paradigm in adaptive thermogenic mechanism has held that heat is generated by uncoupling protein 1 (UCP1) located in the mitochondrial inner membrane of brown or beige adipocytes. During mitochondrial respiration, UCP1 uncouples ATP synthesis and catalyzes proton leak across mitochondrial membrane, resulting in heat generation. However, recent studies discovered UCP1-independent adaptive thermogenic pathways mediated by futile creatine or calcium cycling in mammals (34–36). The futile cycles dissipate energy as the form of heat by consumption of ATP derived from lipid or glucose oxidation and are mainly found in beige adipocytes rather than brown adipocytes due to their high ATP synthetase expression. Like traditional UCP1-dependent thermogenesis, the UCP1-independent thermogenic machinery is also sufficient to regulate whole-body energy homeostasis and protect against diet-induced obesity and related metabolic dysfunction (35–38).

Adaptive thermogenic activation is directed by intercellular crosstalk within adipose niches. Neuronal-thermogenic adipocyte communication through catecholamines is the most well-known. The innervation of sympathetic nervous system (SNS) in thermogenic adipose tissues connects the central nervous system (CNS)-originated efferent signals with brown and beige adipocytes. Upon cold environment, catecholamines, particularly norepinephrine, released from sympathetic nerve terminals activates thermogenic programs of existing brown and beige adipocytes *via* β3-adrenergic receptor (AR) and induce *de novo* beige fat biogenesis *via* β1-AR in rodents (39–42). It is of note that in humans, β2 subtype has been recently reported to play a dominant role in β-ARs-dependent thermogenic fat activation (43). Besides sympathetic innervation or denervation, the local noradrenergic tone within thermogenic adipose tissues has been proposed to be mediated by adipose resident immune cells, such as subpopulations of ATM synthesizing or degrading catecholamine (17, 19). Intriguingly, recent studies also identified cholinergic adipose macrophages (ChAMs) that secrete acetylcholine to selectively activate beige fat thermogenesis under cold exposure (15). Hence, the regulation and function of thermogenic fat need to be understood considering its heterogeneity and complexity stemming from the tissue microenvironment as a bona fide platform for developing thermogenic fat-targeting therapeutic interventions.

## A Classical View of Macrophages in Thermogenic Adipose Niches: Inflammation

Emerging evidence of macrophages’ involvement in controlling thermogenic fat function has recently drawn enormous attention to the non-canonical roles and regulation of ATMs (discussed details in the next section). However, recruitment of pro-inflammatory macrophages in thermogenic adipose tissues has been accepted as a pathophysiological phenomenon of obesity, even though it is less profound than in visceral WAT − the fat depot containing mainly white adipocytes to store energy (14, 44, 45). Chronic inflammation stemming from prolonged calorie overload contributes to the whitening of thermogenic adipocytes that transforms the cells to possess intensive energy-storing unilocular lipid droplets and lose unique characteristic of energy expenditure (14).

Gene expression landscapes of obese mouse BAT revealed enriched expression of markers for macrophages-derived pro-inflammatory cytokines, such as *Tnfα*, C-C motif chemokine ligand (*Ccl*) 2, and *Ccl5* (46, 47). Independent studies showed that the activated pro-inflammatory markers were accompanied by reduced expression of thermogenic genes in obese BAT (14, 48–50). As direct evidence, pro-inflammatory macrophage infiltration and related cytokines, such as TNFα and IL-1β, were seen to inhibit the induction of *Ucp1* expression in response to thermogenic stimuli in mouse thermogenic adipose tissues and differentiated C3H10T1/2 stem cells (14, 51, 52). Genetic deletion of TNF receptors in obese mice led to reduced apoptosis and induced transcriptional activation of *Ucp1* in BAT, suggesting a significant role of TNFα-mediated inflammatory response in thermogenic adipobiology during obesity (53). Besides, TNFα appeared to cause insulin receptor substrate 2-mediated insulin resistance in brown adipocytes, supporting that pro-inflammatory signaling could affect a broad spectrum of metabolic processes beyond thermogenic fat cell activity (54).

M1 macrophage infiltration in subcutaneous WAT in obese mouse models shows deleterious effects on beige thermogenesis. Interestingly, a recent study found a self-sustained cycle of inflammation-driven beiging inhibitory mechanism in which pro-inflammatory macrophages expressing α4 integrin directly interact with beige adipocytes and their precursors through vascular cell adhesion molecule-1 (55). Genetic or pharmacological inhibition of the adhesive interaction enhanced beige adipogenesis and whole-body energy expenditure, thereby attenuating obese phenotype. In addition, genetic ablation of IκB kinase ε that amplifies inflammation signal by its elevation in ATMs enhanced UCP1 expression in subcutaneous WAT of mice (56). Hence, in obesity, recruitment of pro-inflammatory macrophages occurs in thermogenic adipose tissues, as seen in WAT, as a hallmark of obesity and negatively influences the biological functions of brown and beige adipocytes.

## The Adipose Macrophages Central to Adaptive Thermoregulation

Since Nguyen et al. first reported catecholamine-producing ATMs in BAT in 2011, non-classical roles of ATMs in the regulation of thermogenesis and systemic energy homeostasis had been received significant attention and extensively studied as an innovative view to explain complex thermogenic mechanisms (17). To date, the research effort discovered four subpopulations of ATMs that played a direct role in controlling thermogenic fat function and broadened our understanding of the significance of adipose resident immune cells in systemic energy homeostasis.

### Alternatively Activated Macrophages

In contrast to M1 macrophages, M2 or alternatively activated macrophages are predominant in lean adipose tissues and release anti-inflammatory cytokines, such as TGFβ and IL-10. In an obese state, a phenotypic transformation from anti-inflammatory M2 to pro-inflammatory M1-like macrophages occurs in adipose tissues leading to insulin resistance. M2 macrophages are required for tissue repair, tissue homeostasis and anti-helminthic activities. Interestingly, helminth infection attenuated high-fat diet-induced obesity along with induction of adaptive thermogenic capacity through enhanced M2 macrophage polarization (57).

Accumulated evidence shows the association between the expansion of alternatively activated macrophages and thermogenic activation (58–62). Many studies have highlighted how M2 macrophages are polarized and activated during adipose thermogenesis, such as through C-X-C motif chemokine ligand 14 (CXCL14) and meteorin-like (58, 61). Particularly, it is fascinating that CXCL14 is released from brown adipocytes to recruit M2 macrophages upon thermogenic activation, indicating an active interplay between thermogenic adipocytes and M2 macrophages during adaptive thermogenesis. However, the mechanisms by which M2 macrophages induce thermogenic responses have not been well established. Nguyen et al. first identified that upon cold stress M2 macrophages expressing tyrosine hydroxylase (TH), key catecholamine synthesizing enzyme, release norepinephrine *via* IL-4 signaling to activate BAT thermogenesis (17). Serial studies from the same group have found catecholamine-producing M2 macrophage-mediated thermogenesis in beige adipocytes as well and completed its whole mechanistic machinery. In mouse subcutaneous WAT, IL-5 secreted from stimulated type 2 innate lymphoid (ILC2) cells by IL-33 were identified to activate and recruit eosinophils, and subsequently, eosinophils activated M2 macrophages by secreting IL-4 (63, 64). Additionally, ILC2- and eosinophil-secreted type 2 cytokines, including IL-13 and IL-4, promoted beige differentiation of PDGFRα^+^ precursors through IL-4Rα (64). However, in 2017, Fischer et al. presented contradictory data that M2 macrophages do not express TH enough to produce norepinephrine in BAT and subcutaneous WAT (20). It is still controversial whether alternatively activated macrophages synthesize NE during thermogenesis based on published independent studies (60, 65–67). It has been advances in identifying thermogenic ligands for brown and beige adipocytes within adipose niches. However, whether alternatively activated macrophages can produce any known ligands other than NE for thermogenic fat activation is still unclear (68).

A recent study provided a novel aspect of M2 macrophage-dependent catecholamine secretion and beiging by discovering the mechanism by which M2 macrophages enhanced the local SNS activation in subcutaneous WAT upon cold exposure. Slit guidance ligand 3 (SLIT3) secreted from M2 macrophages induced sympathetic innervation and TH activation by binding to sympathetic neurons *via* roundabout guidance receptor 1 (ROBO1), thereby promoting NE synthesis and beiging to sustain adaptive thermogenesis (16). Consistent with this notion, an independent study found that IL-25-induced M2 macrophage polarization increased outgrowth of sympathetic nerves in subcutaneous WAT (60). It is conceivable that M2 macrophages contribute to adaptive thermogenesis, and dissecting the mechanisms by which M2 macrophages induce thermogenesis and maintain systemic energy homeostasis may empower the specific immune cell type as a novel therapeutic target for obesity.

### Sympathetic Innervation-Regulatory Macrophages

Under a cold environment, TH-expressing sympathetic axons are the primary source of NE that binds to β3-ARs on the surface of mature brown and beige adipocytes and activates thermogenic programs. A recent mouse study indicated that beige adipocytes could be differentiated from αSMA-expressing progenitors through β1-ARs in response to NE (39). Of note, beige adipocytes are greatly inducible and heterogeneous due to their remarkable plasticity at the adipocyte and progenitor levels in response to external cues or genetic disposition. In mice lacking β-ARs, beige adipocytes were activated by directly sensing cold temperature or differentiated from myogenic differentiation 1-expressing progenitors (known as glycolytic beige adipocytes) (40, 69).

Local sympathetic innervation and activity in rodent BAT and subcutaneous WAT are enhanced by adipocyte-derived neurotrophic factors, such as nerve growth factor (NGF), neuregulin 4 and S-100 protein β-chain, and by vascular endothelial growth A factor secreted from vascular cells and brown adipocytes (70–74). Recent studies highlight the contribution of immune cells resident in thermogenic adipose niches to sympathetic innervation under cold conditions. In mouse BAT, γδ T cells induced TGFβ1 secretion from adipocytes through IL-17 receptor C signaling and thus increased outgrowth of sympathetic nerves and adaptive thermogenesis (75). Eosinophils maintained by IL-5 also secreted NGF, thereby promoting sympathetic innervation during cold-induced beiging (76).

Methyl-CpG binding protein 2 (*Mecp2*)-expressing, CX3CR1^+^ macrophages have been suggested as a subpopulation of BAT-resident macrophages that directly influences sympathetic innervation (18). *Mecp2* is well-known to show a mutation in the postnatal neurodevelopmental disorder Rett syndrome. Both *Mecp2*-null and brain-restricted *Mecp2*-deleted mice demonstrated neurological defects related to Rett syndrome, such as irregular breathing and hindlimb clasping (77). Wolf et al. genetically depleted *Mecp2* in all tissue macrophages using *Cx3cr1-Cre* mice or in a macrophage subpopulation expressing CX3CR1 using tamoxifen-inducible *Cx3cr1-CreER* mice (18, 78). Upon chow diet feeding, the mutant mice developed obese phenotypes with excessive body weight gain and enlarged fat mass without Rett-like symptoms in adulthood (18). Thermogenic dysfunction of BAT and resulted decrease in whole-body energy expenditure at a steady-state were seen in the mutant animals due to reduced NE input by impaired local sympathetic innervation. Mechanistically, Plexin A4 overexpressed in *Mecp2*-deleted CX3CR1^+^ macrophages seemed to interact with and block outgrowth of Semaphorin 6A-positive sympathetic axons in the tissue.

Interestingly, *Mecp2-*expressing macrophages were found to be insignificant in coordinating the responsiveness to acute thermogenic stimulation, such as brief cold exposure (18). Instead, it was linked to sustaining sympathetic innervation and adaptive thermogenesis at a steady-state for homeostasis. As discussed, alternatively activated macrophages have also been reported to involve sympathetic nerve outgrowth in subcutaneous WAT during cold adaptation (16, 60). Therefore, available evidence indicates that a discrete subpopulation of ATMs may reshape local sympathetic innervation to mediate energy expenditure and maintain metabolic homeostasis.

### Catecholamine-Scavenging Macrophages

Over the controversy surrounding the presence and significance of catecholamine-producing macrophages within thermogenic adipose tissues, Pirzgalska et al. identified a subpopulation of ATMs that contains catecholamine by its uptake, not by its biosynthesis, in both rodents and humans and that mediates thermogenesis and obesity (19). The macrophage subset, named sympathetic neuron-associated macrophages (SAMs), was initially found in sympathetic nerve bundles of subcutaneous WAT and confirmed its existence in BAT macrophages in mice. SAMs have been characterized as hematopoietic lineage cells that have enriched expression of macrophage-specific markers and as a functionally distinct population that uniquely expresses solute carrier family 6 member 2 (*Slc6a2*) responsible for NE transport. NE was detectable in SAMs, and the NE uptake depended on extracellular catecholamine availability. Additionally, the absence of TH in SAMs supported the notion that intercellular NE accumulation was due to its uptake, not biosynthesis. To coordinate the cellular catecholamine scavenging process, SAMs also highly expressed monoamine oxidase A (*Maoa*) for NE degradation along with *Slc6a2*. Previous studies also prove that macrophages are capable of NE uptake and degradation (79, 80). Interestingly, beige adipocytes served as a NE clearance route *via* organic cation transporter 3 (*Oct3*) in subcutaneous WAT, and fat-specific *Oct3*-deleted mice showed enhanced beige thermogenesis in response to NE (81).

Adipose SAMs have been found to be excessively recruited in obese conditions, suggesting their possible role in pro-inflammation (19). However, when SAM-mediated catecholamine uptake was blocked by bone-marrow transplantation from *Slc6a2* KO mice into obese recipients, the chimeric mice showed elevated serum NE levels and adaptive thermogenic capacity in both BAT and subcutaneous WAT under cold exposure. Furthermore, SNS activation *via* food restriction resulted in body weight loss in the chimeric animals due to activation of adipocyte lipolysis, the first step to generate energy substrates for thermogenesis in thermogenic adipocytes. An independent study uncovered a *Maoa*-enriched macrophage subset within visceral WAT (82). The distinct subpopulation was found to lower the bioavailability of NE and blunt adipocyte lipolysis in the elderly. Mechanistically, its catecholamine-degradation machinery was activated by NLRP3 inflammasome-induced growth differentiation factor 3 (82).

This potential integrative explanation involving the coexistence of catecholamine scavenging and producing ATM subpopulations within thermogenic adipose niches indicates the significance of ATMs in maintaining local noradrenergic tone and related metabolic homeostasis. Furthermore, the catecholamine scavenging ATMs may also play a role in regulating systemic NE homeostasis upon sustained hypernoradrenergic conditions.

### Cholinergic Adipose Macrophages (ChAMs)

Acetylcholine is arguably accepted as the most crucial neurotransmitter in the CNS, autonomic nervous system and somatic nervous system. Parasympathetic nerves are the primary source of acetylcholine. However, non-neuronal cell types also contain cellular machinery that modulates acetylcholine availability and utilization, called the non-neuronal cholinergic system (NNCS), to maintain physiological functions and homeostasis of key organs (83–85). Furthermore, dysfunctional NNCS is directly linked to the pathogenesis of diseases (84, 85). Hence, identifying and understanding NNCS in various types of cells provides new insights to treat diseases.

NNCS includes an acetylcholine synthesizing enzyme [choline acetyltransferase (ChAT)], transporters [vesicular acetylcholine transporter (VaChT)], receptors [nicotinic acetylcholine receptors (nAChRs), muscarinic acetylcholine receptors (mAChRs)] and degrading enzymes [acetylcholinesterase, (AChE), butyrylcholinesterase (BChE)] (84, 85). Immune cells have been known to express NNCS components, particularly ChAT. Recent findings highlight distinct biological roles of acetylcholine-synthesizing immune cells, particularly T cells and B cells, in maintaining innate immunity and blood pressure and responding to chronic viral infection (86–88).

ChAT has been reported to be expressed in macrophages; however, the physiological significance of the acetylcholine-producing macrophages has not been elucidated (86, 89–91). Jun et al. recently identified ChAMs responsible for acetylcholine secretion and adaptive thermogenesis in subcutaneous WAT where parasympathetic innervation is absent (15, 92, 93). Using ChAT reporter mice to monitor functional ChAT-expressing cells *in vivo*, acetylcholine-producing cell populations were defined as CD45^+^ hematopoietic immune cells, not neurons, and they were composed mainly of B cells, T cells and macrophages (92). Among the ChAT^+^ immune subsets, macrophages were the only population to show induction in ChAT abundance and acetylcholine secretion in response to cold stress (15). Macrophage-specific ChAT deleted mice showed impaired cold-induced thermogenesis in subcutaneous WAT. ChAMs were activated by NE through β2-AR signaling, demonstrating their role in linking sympathetic signals to thermogenic beige fat activation (15). As suggested by transcriptome analysis, the functionally distinct ChAMs displayed unique molecular machinery fulfilling their role in neurotransmitter metabolism (15).

It has been found that neuronal acetylcholine receptor subunit alpha 2 (CHRNA2) senses ChAMs-produced acetylcholine in beige adipocytes and activates thermogenesis through UCP1- and creatine futile cycling-mediated pathways (92, 94). CHRNA2 has been identified as a unique marker for activated beige adipocytes. However, brown adipocytes do not have functional CHRNA2 to receive acetylcholine (92). Besides, the abundance of ChAT^+^ macrophages in BAT was very low and not responsive to cold exposure (15). Therefore, CHRNA2-dependent thermogenic activation was not seen in BAT. CHRNA2 signaling was induced during cold exposure, and its deficiency at the whole-body or adipocyte level in mice was unable to fully activate cold-induced thermogenic programs in subcutaneous WAT and systemic energy expenditure (92, 94). Importantly, this acetylcholine-mediated CHRNA2 signaling showed physiological significance to combat obesity and related metabolic dysfunction by demonstrating profound fat accumulation and impaired whole-body glucose metabolism after chronic calorie overload in the absence of *Chrna2*. The discovery of ChAMs that secrete acetylcholine *via* β2-AR signaling and induce beige thermogenesis provides novel evidence of the neuro-immune-beige fat axis and may offer a new approach to counteract obesity and metabolic disorders.

## Concluding Remarks

**Figure 1:**
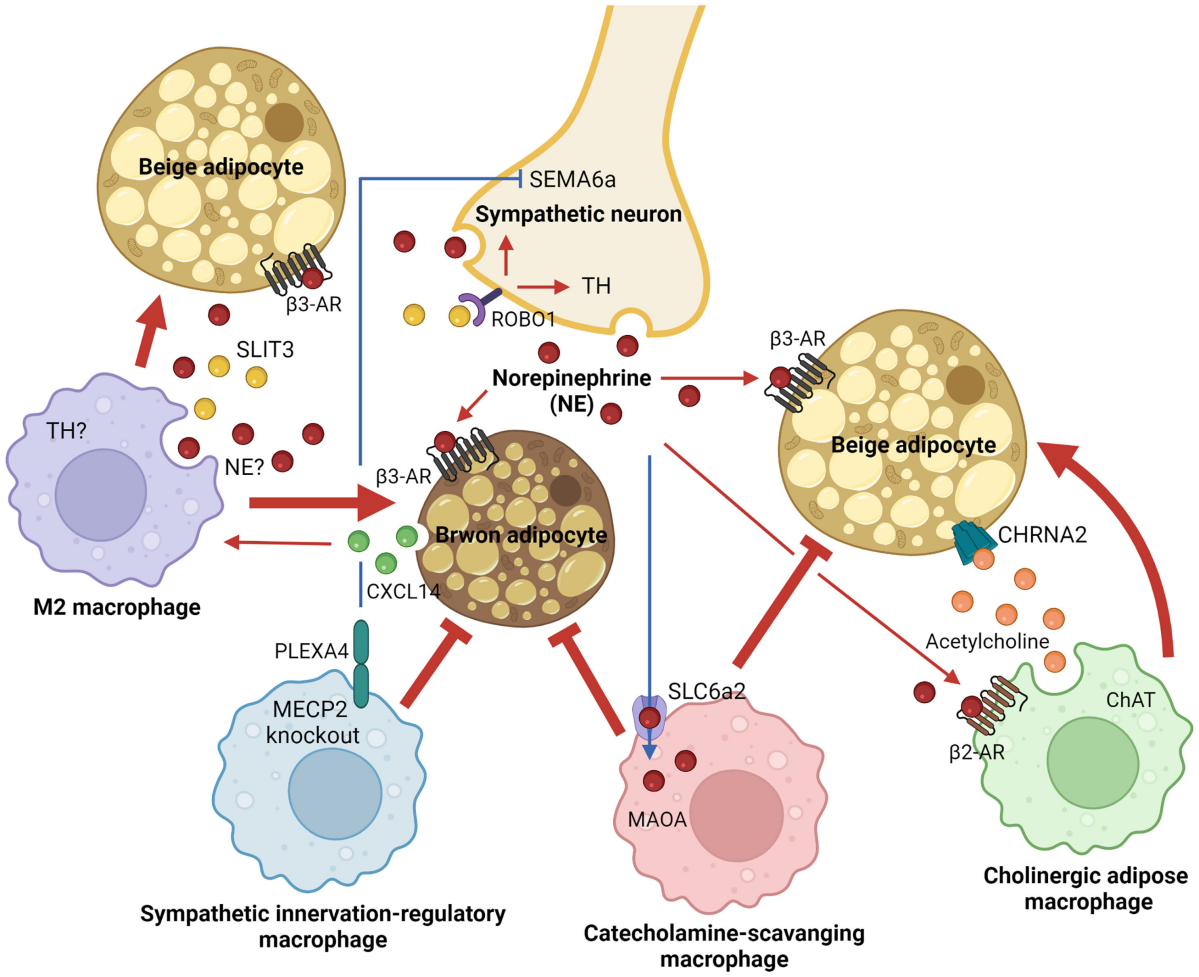
Adipose tissue macrophage-mediated adaptive thermogenesis in brown and beige adipocytes. Distinct macrophage subpopulations within thermogenic adipose tissues, including brown and subcutaneous white fat, support functions of brown or beige adipocytes to dissipate energy and regulate systemic energy homeostasis. Alternatively activated M2 and cholinergic adipose macrophages activate thermogenic responses in brown or beige adipocytes through paracrine mechanisms in a cold environment (red arrows). On the other hand, thermogenic inhibitory ATM subsets that block sympathetic innervation or import/degrade catecholamine have been identified in thermogenic adipose niches (red blunt-ended lines). AR, adrenergic receptor; ChAT, choline acetyltransferase; CHRNA2, neuronal acetylcholine receptor subunit alpha 2; CXCL14, C-X-C motif chemokine ligand 14; MAOA, monoamine oxidase A; MECP2, methyl-CpG binding protein 2; NE, norepinephrine; PLEXA4, plexin A4; ROBO1, roundabout guidance receptor 1; SEMA6a, semaphoring 6a; SLC6a2, solute carrier family 6 member 2; SLIT3, slit guidance ligand 3; TH, tyrosine hydroxylase.

Thermogenic fat in adult humans was first found and described as the interscapular gland in 1908 (95). Around a hundred years later, its rediscovery, in which thermogenic fat is inducible and functional in adult humans upon external stimuli, fueled scientific interest in the field and contributed to recent advances in understanding thermogenic adipobiology (21–23). Over the last decade, immune-thermogenic adipose interaction has received particular attention in the field due to its unexpected critical roles in shaping thermogenic fat function. Macrophages have been identified as a major resident immune cell type in thermogenic adipose niches. Four distinct subsets of ATMs central to thermoregulation have been identified so far, as summarized in **Figure 1**. Secretory response to cold exposure was seen in both M2 macrophages and ChAMs to rapidly stimulate thermogenic machinery in a paracrine manner. Surprisingly, M1 macrophages-derived inflammatory cytokine CXCL5 has been reported to induce beige thermogenesis (96). On the contrary, there are ATM subsets that inhibit adaptive thermogenesis and exert pathogenesis of obesity. The four ATM subpopulations have been characterized as distinct populations that express their unique markers or cellular machinery. However, it is still unknown whether they share their molecular natures or developmental origins, at least in part, and interact with each other in a particular environment. In addition, their distribution across tissues and tissue-specific functions is worth studying to understand their biological significance better at the whole-body level.

The unexpected discovery of acetylcholine-secreting ChAMs in subcutaneous WAT raises the possibility that existing distinct macrophage subpopulations within thermogenic adipose niches might be more diverse than we think. ChAMs are the only ATM subset so far known to target a non-canonical thermogenic pathway, CHRNA2 signaling, in beige adipocytes among the four thermogenic ATMs. In addition, ChAMs responded to β2-AR agonist or fibroblast growth factor 21 mediated by miRNA-182-5p from beige adipocytes to induce acetylcholine secretion in a cold environment (15, 97). The other three ATM subpopulations are linked to the canonical β-AR mechanism in thermogenic adipocytes *via* regulating local noradrenergic tone. Further work studying mechanistic details that modulate activation and target thermogenic pathways of the ATM subsets may contribute to defining their distinct features and functions from each other in regulating adipose thermogenesis and metabolism. For example, it is reasonable to question whether and how the thermoregulatory ATMs’ functions are mediated by the dynamic regulators of macrophage activation that control cytokine secretion and innate immunity in response to external cues and also affect the recruitment of subtypes of thermogenic adipocytes (98–100). In addition, since chronic excessive systemic acetylcholine or catecholamine causes disease conditions, such as acetylcholine-induced nicotinic/muscarinic toxicity and catecholamine-induced hypertension, we need to investigate whether their levels mediated by the thermogenic ATM subsets are limited at the local site level or affect systemic levels (101–103). Furthermore, these ATM subsets’ presence and biological significance need to be assessed in human thermogenic adipose tissues.

There is growing awareness that brown and beige adipocytes improve lipid metabolism, glucose metabolism and insulin sensitivity beyond enhanced energy expenditure. However, underlying regulatory mechanisms of thermogenic adipocytes are complex due to their remarkable plasticity and heterogeneity depending on external cues, genetic disposition and microenvironment. Although understanding of adaptive thermogenic mechanisms still remains incomplete, discovering the ATM subpopulations central to thermoregulation has broadened our knowledge of immune-thermogenic adipose interaction in metabolic adaptation and brought new insights into the development of therapeutic strategies to enhance energy expenditure. Further research in dissecting ATM populations to identify a specific subset that supports the functions of thermogenic adipocytes may provide exciting perspectives in the field and establish an immune-targeting metabolic drug to improve systemic energy homeostasis and reverse the pathophysiology of obesity and other metabolic disorders.

## Author Contributions

HJ structured the manuscript and made the figure. MR and HJ reviewed literature and wrote the manuscript. All authors contributed to the article and approved the submitted version.

## Funding

This work was supported by the College of Human Sciences, Texas Tech University.

## Conflict of Interest

The authors declare that the research was conducted in the absence of any commercial or financial relationships that could be construed as a potential conflict of interest.

## Publisher’s Note

All claims expressed in this article are solely those of the authors and do not necessarily represent those of their affiliated organizations, or those of the publisher, the editors and the reviewers. Any product that may be evaluated in this article, or claim that may be made by its manufacturer, is not guaranteed or endorsed by the publisher.
